# Detection of IgG Antibodies Against COVID-19 N-Protein by Hybrid Graphene–Nanorod Sensor

**DOI:** 10.3390/bios15030164

**Published:** 2025-03-04

**Authors:** R. V. A. Boaventura, C. L. Pereira, C. Junqueira, K. B. Gonçalves, N. P. Rezende, I. A. Borges, R. C. Barcelos, F. B. Oréfice, F. F. Bagno, F. G. Fonseca, A. Corrêa, L. S. Gomes, R. G. Lacerda

**Affiliations:** 1 Physics Department, Federal University of Minas Gerais (UFMG), Belo Horizonte 31270, MG, Brazil; renatoveloso1803@ufmg.br (R.V.A.B.); cpereira@fisica.ufmg.br (C.L.P.); kennedybatista@ufmg.br (K.B.G.); nataliarezende@ufmg.br (N.P.R.); forefice@ufmg.br (F.B.O.); liviasg@fisica.ufmg.br (L.S.G.); 2 CTNano, Federal University of Minas Gerais (UFMG), Belo Horizonte 31270, MG, Brazil; caroline.junqueira@ctnano.org (C.J.); iara.borges@ctnano.org (I.A.B.);; 3 Microbiology Department, Federal University of Minas Gerais (UFMG), Belo Horizonte 31270, MG, Brazil; fdafonseca@icb.ufmg.br; 4 Chemistry Department, Federal University of São João del-Rei (UFSJ), Divinópolis 35500-008, MG, Brazil; rosicbarcelos@ufsj.edu.br; 5 Vaccine Technology Center (CT Vacinas), BH-Tec, UFMG, Belo Horizonte 31270, MG, Brazil; flaviabagno@ctvacinas.org

**Keywords:** graphene, biosensor, transistor, gold nanorod

## Abstract

The COVID-19 pandemic highlighted the global necessity to develop fast, affordable, and user-friendly diagnostic alternatives. Alongside recognized tests such as ELISA, nanotechnologies have since been explored for direct and indirect diagnosis of SARS-CoV-2, the etiological agent of COVID-19. Accordingly, in this work, we report a method to detect anti-SARS-CoV-2 antibodies based on graphene-based field-effect transistors (GFETs), using a nanostructured platform of graphene with added gold nanorods (GNRs) and a specific viral protein. To detect anti-N-protein IgG antibodies for COVID-19 in human sera, gold nanorods were functionalized with the nucleocapsid (N) protein of SARS-CoV-2, and subsequently deposited onto graphene devices. Our test results demonstrate that the sensor is highly sensitive and can detect antibody concentrations as low as 100 pg/mL. Using the sensor to test human sera that were previously diagnosed with ELISA showed a 90% accuracy rate compared to the ELISA results, with the test completed in under 15 min. Integrating graphene and nanorods eliminates the need for a blocker, simplifying sensor fabrication. This hybrid sensor holds robust potential to serve as a simple and efficient point-of-care platform.

## 1. Introduction

COVID-19 is an infectious disease caused by severe acute respiratory syndrome coronavirus 2 (SARS-CoV-2), a highly transmissible pathogen first identified in China in December 2019 [1]. On 11 March 2020, the World Health Organization declared COVID-19 a pandemic [2]. The clinical symptoms of COVID-19, including fever, dry cough, fatigue, and anosmia, were initially documented within the first week of infection in early patient cohorts [3]. To mitigate viral transmission, public health measures such as mask-wearing, social distancing, and frequent hand disinfection were widely implemented. Concurrently, the urgent need for rapid diagnostic tests and effective vaccines became a global priority [4].

Coronaviruses are spherical, enveloped viruses measuring 70–90 nm in diameter, with a single-stranded RNA genome of positive polarity (ssRNA+) [5]. The viral envelope is composed of three structural proteins: spike (S), membrane (M), and envelope (E). Encased within the nucleocapsid are multiple copies of the nucleocapsid (N) protein, along with the viral RNA genome [5,6].

During the development of vaccines and diagnostic assays, the S-protein emerged as a primary target, due to its receptor-binding domain (RBD), which facilitates viral entry into host cells. Consequently, it became the principal target for neutralizing immune responses [7]. However, the emergence of SARS-CoV-2 variants has led to mutations predominantly affecting the S-protein, compromising the sensitivity of S-protein-based diagnostic tests. In contrast, the N-protein exhibits lower mutational variability, while eliciting a robust humoral response, making it an attractive target for diagnostic and vaccine development [8].

Accurate diagnosis is critical for controlling the spread of infectious diseases, by confirming pathogen presence and enabling epidemiological surveillance. Enzyme-linked immunosorbent assay (ELISA) is the gold-standard serological test for detecting anti-SARS-CoV-2 IgG antibodies [9,10,11,12]. However, ELISA has notable drawbacks, including labor-intensive antibody preparation, high costs due to technical complexity, susceptibility to false positives or negatives from inadequate surface blocking [13], and a prolonged assay time of at least 75 min [14].

Beyond ELISA, lateral flow immunoassay (LFIA) is widely utilized for rapid SARS-CoV-2 antibody detection in human blood via immunochromatographic techniques [15]. Despite providing results within 10–15 min, LFIA exhibits a higher incidence of false positives and negatives compared to ELISA [16]. To overcome these limitations, the development of point-of-care diagnostic platforms that maintain high accuracy while eliminating the need for laboratory environments remains imperative.

Graphene has emerged as a highly promising material for biosensing applications, due to its extraordinary properties, including high electronic mobility, a large monolayer thickness, exceptional conductivity, and high biocompatibility [17,18,19]. Its high surface-to-volume ratio and functionalization capabilities enable the detection of charge transfer events from biochemical interactions, which can be transduced into electrical signals [20,21,22].

Recent studies have demonstrated that graphene-based sensors can be optimized by incorporating nanoparticles, such as metallic, oxide, and semiconductor nanomaterials, into graphene sheets to create hybrid graphene–nanoparticle structures [23]. Functionalization of graphene with noble metal nanoparticles has been extensively explored to enhance intrinsic properties and introduce novel functionalities. Hybrid structures of noble metal nanoparticles and graphene offer great potential for label-free biosensing applications [24,25,26]. Unlike linker molecules such as pyrene [27] and diazonium [28], noble metal nanoparticles enable rapid, efficient, and straightforward immobilization of functionalized receptor molecules on biosensors through strong covalent bonds, such as Au–S [29].

Graphene-based biosensors employ various sensing mechanisms, including optical, electrochemical, and electrical detection. The optical transparency of graphene enhances plasmonic sensor performance, while chemically derived graphene derivatives exhibit edge-plane-like defects that facilitate electron transfer [30]. Additionally, graphene oxide (GO) exhibits strong fluorescence quenching, enabling fluorescence resonance energy transfer (FRET)-based biosensors [31]. Graphene has also been successfully integrated into surface-enhanced Raman spectroscopy (SERS) platforms, leveraging its ability to induce strong chemical enhancement [32].

For electrochemical biosensing, graphene-based field-effect transistors (G-FETs) offer high electron transfer rates, exceptional charge carrier mobility, and low electrical noise, enabling the sensitive detection of biomarkers in biological samples such as serum and blood [33,34]. This approach provides high detection sensitivity and near-instantaneous results.

Several research groups have developed G-FET biosensors for SARS-CoV-2 detection, with devices successfully detecting the S-protein in nasopharyngeal swabs within minutes [35,36,37,38,39,40,41], achieving detection limits as low as 1 fM [36]. Additionally, G-FET biosensors have demonstrated the ability to detect anti-S-protein IgG antibodies in human serum [42,43,44,45,46], with a reported limit of detection (LOD) for monoclonal IgG as low as 1 pg/mL [34]. However, given the high mutation rate of the S-protein, these biosensors may exhibit reduced sensitivity across viral variants. To address this limitation, researchers have developed sensors that can directly detect the N-protein [45,46]. Nevertheless, no studies have reported the development of G-FET biosensors for detecting anti-N-protein antibodies.

**Figure 1:**
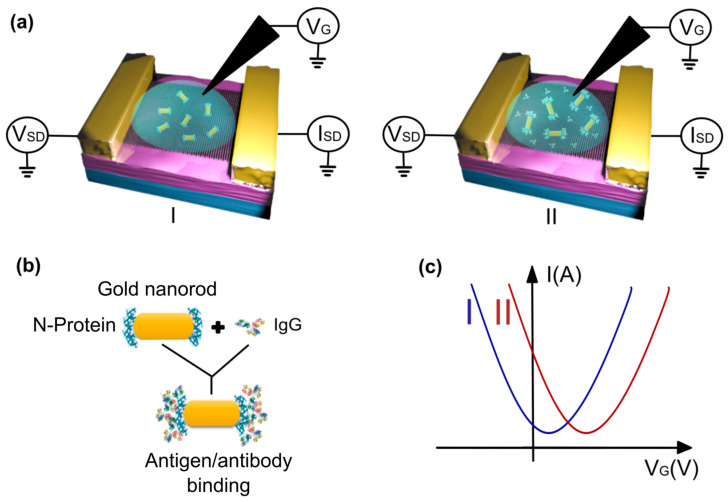
(**a**) A schematic model of the process of detecting the binding between the N-protein and the IgG antibody using the graphene field-effect transistor (GFET) platform, before (I) and after (II) the addition of human serum. (**b**) The antigen/antibody binding process, wherein the IgG antibody links to the N-protein at the edges of the gold nanorod. (**c**) A model of transfer curves before (I) and after (II) the binding, showing p-type doping in graphene.

In this study, we developed a G-FET biosensor for detecting anti-N-protein IgG in human serum (Figure 1a). To facilitate IgG detection, gold nanorods, functionalized with SARS-CoV-2 N-protein at their extremities (Figure 1b), were deposited onto the graphene surface. This hybrid graphene–nanorod system eliminated the need for blocking agents, simplifying the biosensor fabrication process. Binding of the anti-N-protein IgG and the N-protein led to a charge transfer on the graphene surface, which was discerned through variations in the GFET transfer curve (Figure 1c) [34]. Using a monoclonal anti-N-protein IgG antibody, our biosensor achieved an LOD of 100 pg/mL. Moreover, as a proof of concept, clinical tests conducted using human serological samples demonstrated a success rate of 90% in comparison to ELISA, with results achieved in less than 15 min, providing robust evidence of the potential application of our hybrid sensor for point-of-care diagnostics.

## 2. Materials and Methods

The GFET chip was fabricated following the methodology detailed in our previous reports [47,48,49]. Graphene was obtained through mechanical exfoliation of graphite, and subsequently transferred onto a SiO_2_ substrate. Gold/chrome (30 nm/1 nm) electrical contacts were patterned using electron beam lithography, and deposited via thermal evaporation.

Gold nanorods (GNRs) were synthesized using a seed-mediated growth method, and subsequently surface-activated [50]. Transmission electron microscopy (TEM) was used for the morphological characterization of the GNRs, as shown in Figure S1. Recombinant SARS-CoV-2 N-protein was provided by Dr. Flávio G. da Fonseca from the Vaccine Technology Center (CTVacinas) at the Federal University of Minas Gerais (UFMG). The production and purification procedures have been previously described [12]. Protein batches were received in 400 μL volumes, at a concentration of 1.1 mg/mL.

For bioconjugation, the N-protein was covalently linked to the extremities of GNRs using 11-Mercaptoundecanoic acid (MUA, 95%, Sigma-Aldrich, St. Louis, MO, USA) as a coupling agent. The carboxyl groups of MUA were activated with N-ethyl-N’-(3-dimethylaminopropyl) carbodiimide (EDAC) and N-hydroxy succinimide (NHS), to enable binding to the N-protein’s amine groups. The thiol group of MUA formed a stable bond with the gold surface of the nanorods. To demonstrate the functionalization of GNRs, zeta potential measurements were performed for GNRs, GNRs plus MUA, and GNRs/MUA with N-proteins. The zeta potential reflects the surface charge of suspended particles, and is directly linked to colloidal stability. Additional details regarding GNR–N-protein conjugation can be found in Supplementary Data S1 and Figure S2.

A 10 μL aliquot of bioconjugated GNR solution (B-GNR, 1 nM) was carefully deposited onto the graphene surface, followed by spin coating at 2500 rpm for one minute. A polydimethylsiloxane (PDMS) well with a central aperture was affixed to the device, to confine liquid exposure to the graphene region. Phosphate-buffered saline (PBS, 0.01×) was used as the electrolyte solution, yielding a Debye length of 7.3 nm [51].

SARS-CoV-2 monoclonal antibodies (MERCK) were diluted in 0.01× PBS to various concentrations (100 pg/mL, 1 ng/mL, 10 ng/mL, and 1 μg/mL) for sensitivity analysis. Human serum samples were collected and diagnosed for anti-SARS-CoV-2 IgG using a standardized and validated ELISA protocol [12]. Sera were diluted at 1:1000 in 0.01× PBS for subsequent analysis.

Raman spectra and hyperspectral Raman maps were acquired using a WITec Alpha300R system (Oxford Instruments, Abingdon, UK) with a 532 nm excitation laser (0.8 mW power). The hyperspectral maps consisted of 289 spectra covering a 10 μm × 10 μm area on graphene. Surface roughness was analyzed via atomic force microscopy (AFM) using a Solver Nano instrument (NT-MDT Spectrum Instruments). Scanning electron microscopy (SEM) images were captured to verify B-GNR deposition (Figure S3). Further details on SEM analysis are provided in Supplementary Data S2.

Electrical measurements were conducted by applying a 0.1 V potential between the source and drain terminals (VSD) using a Stanford Research Systems SR830 lock-in amplifier. The resulting current (I) was recorded with a Keithley 2000 multimeter. An Ag/AgCl commercial electrode (eDAQ) immersed in PBS served as the electrochemical gate (VG), which was varied from −0.8 V to 0.8 V. Additional details on the Ag/AgCl electrode can be found in Supplementary Data S3. The detected electrical response signal was normalized as [ΔI/I_0_] = [(I − I_0_)/I_0_] × 100%, where I represents the real-time detected current, and I_0_ is the initial current at 0.1 V, to the right of the charge neutrality point (CNP) in the first transfer curve after the conditioning process. For clarity, Figure S4 illustrates this procedure. All measurements were performed at ambient temperature and pressure.

## 3. Results

**Figure 2:**
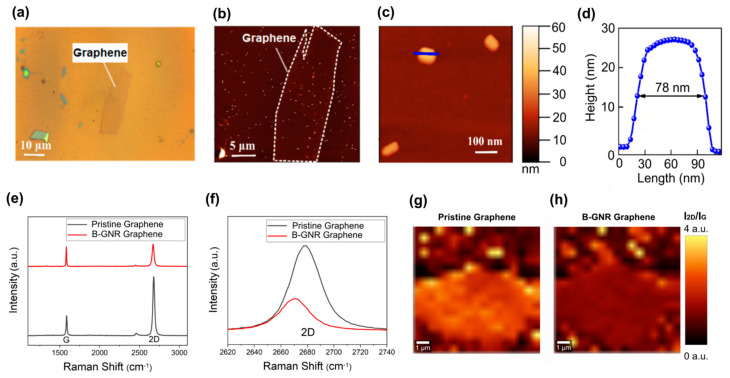
The biosensor characterization was conducted using AFM and Raman spectroscopy. (**a**) An optical image depicts a graphene monolayer after the deposition of gold nanorods. (**b**) An AFM topographic image of the same graphene is presented, delineated by a dashed line, with several deposited nanorods visible as light dots in the image. (**c**) An AFM image shows three isolated nanorods, with the same topographic scale as in b and c. (**d**) A height profile of a nanorod is included, with the line indicated in c, revealing an average height of 27 nm and a length of approximately 78 nm. (**e**) Raman spectra of graphene before and after B-GNR deposition are compared. (**f**) A band shift from the 2D band in e illustrates n-type doping. (**g**) A spectral map of I_2D_/I_G_ on pristine graphene is shown. (**h**) A spectral map of I_2D_/I_G_ on B-GNR graphene is also provided.

Atomic force microscopy (AFM) images were acquired to assess the distribution of gold nanorods (GNRs) on the graphene surface in the GFET devices. The AFM analysis revealed a continuous and homogeneous distribution of GNRs, with an average height of 27 nm and a length of 78 nm (Figure 2). This uniform dispersion is crucial for ensuring consistent electrical properties across the device.

Raman spectroscopy was employed to investigate the electronic interaction between graphene and bioconjugated GNRs (B-GNRs). A shift in the 2D band of graphene was observed following B-GNR deposition, indicative of doping effects. The direction of this shift was correlated with alterations in charge carrier density [52,53]. Figure 2f illustrates a redshift in the 2D band, signifying n-type doping due to the interaction between graphene and gold nanorods. Additionally, doping effects were confirmed by examining the intensity ratio of the 2D and G bands (I_2D_/I_G_), where lower ratios correspond to higher doping levels [54]. Figure 2g,h present spectral maps of I_2D_/I_G_ across the graphene surface, further confirming the doping effect.

**Figure 3:**
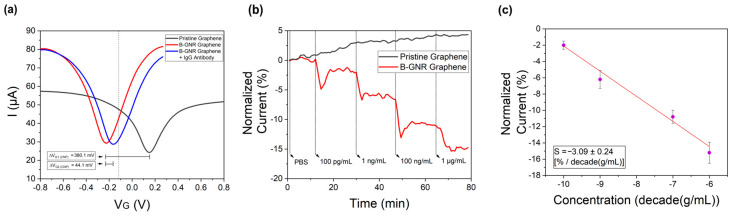
(**a**) The measurements of the transfer curves, with V_SD_ = 0.1 V, for pristine graphene (black line), after B-GNR solution deposition on graphene (red line), and after antigen/antibody binding (blue line). (**b**) The variation in the normalized current at a series of concentrations (100 pg/mL~1 μg/mL) of the target monoclonal IgG antibody in pristine graphene (black line) and in B-GNR graphene, for different concentrations of target IgG monoclonal antibodies (red line). (**c**) A calibration curve for the series of target monoclonal IgG concentrations (semi-log scale). The error bars indicate standard deviation based on measurements with different devices.

Electrical measurements were performed to compare pure graphene with graphene decorated with bioconjugated gold nanorods (B-GNRs). Figure 3a shows the transfer curves recorded with a constant source–drain voltage (V_SD_) of 0.1 V. The black line is related to the measurements on pristine graphene, while the red line represents graphene after decoration with B-GNRs. A leftward shift in the transfer curve was observed, resulting in a change in the charge neutrality point potential (ΔV_G1 (CNP)_) of 380.1 mV. This shift indicates n-type doping of the graphene, consistent with Raman spectroscopy results [55]. The nanorods act as electron donors to graphene, contributing to the n-type doping. This doping effect can also be attributed to the electron-donating nature of biological molecules, particularly due to their nitrogenous groups. Additionally, gold nanoparticles themselves are known to donate electrons to graphene [50]. We believe that the linking between the gold nanorods and graphene is performed via the cetyltrimethylammonium bromide (CTAB) molecules which decorate their lateral surfaces (Figure S5). CTAB is a surfactant that should be removed from GNPs upon contact with water. However, when CTAB is used to link GNPs to graphene, it remains on the surface, even after the washing process (Figure S3). This interaction is primarily driven by Van der Waals forces between the alkyl chains of CTAB and the graphene surface. This is similar to other molecules found in the literature [56].

Subsequent exposure of the functionalized GFETs to IgG monoclonal antibodies (anti-N-protein) in PBS resulted in a rightward shift in the transfer curve (blue line), corresponding to p-type doping. The observed charge neutrality point shift (ΔV_G2 (CNP)_) of 44.1 mV indicates successful antibody binding to the B-GNR-functionalized graphene surface. The specificity of this antigen–antibody interaction serves as an effective biosensing mechanism [22].

For the current versus time graph (Figure 3b), the gate voltage (V_G_) was set to −0.13 V, just 0.1 V to the right of the charge neutrality point (CNP) of the red transfer curve, as indicated by the dashed line in Figure 2a. The variation in current over time was measured and is illustrated in Figure 3b. As the entire transfer curve shifts to the right, the selected point moves closer to the CNP, explaining the observed reduction in current. The transfer curves related to the detection of monoclonal antibodies at different concentrations are provided in Figure S6.

In Figure 3b, it can be seen that the exposure of pristine graphene (black line) to monoclonal antibodies at varying concentrations resulted in a progressive change in electrical current, with each concentration incrementally affecting the signal. The cumulative response exhibited an ~5% variation in normalized current following exposure to all concentrations, consistent with previously reported measurements of antibody interactions with pristine graphene [24]. In contrast, measurements for B-GNR graphene (red line) revealed a rapid and pronounced decrease in electrical current upon antibody exposure, culminating in a ~15% current variation after exposure to all concentrations. These findings established a limit of detection (LOD) of 100 pg/mL for the graphene/nanorod biosensor. To further validate this LOD, additional measurements were conducted (Figure S7) by sequentially introducing PBS and monoclonal antibodies at 100 pg/mL, confirming the sensor’s detection capability.

The experiment involving B-GNR graphene exposure to monoclonal antibodies was repeated several times. Using the resulting data, a semi-logarithmic plot of concentration versus normalized current was generated, as shown in Figure 3c. Sensor sensitivity (S) is defined as the slope of the output (percentage change in normalized current) relative to the input quantity, such as concentration. This sensitivity is derived from the slope of the least-squares fit line in the graph [40]. The calculated sensitivity, S= −3.09 ± 0.24 [% per decade (g/mL)], demonstrates a linear relationship between the current and the logarithmic IgG concentration. The error bars in Figure 3c represent the variation observed across multiple devices during repeated experiments.

Subsequently, electrical measurements were conducted with naturally positive and negative human sera. The potential applied to the source/drain channel (V_SD_) was held constant at 0.1 V, while the potential applied to the gate channel (V_G_) was varied from approximately ±0.25 V from the initial CNP, and the current was monitored accordingly. Under these conditions, diluted serum (1 μg/mL in PBS) was introduced into the electrochemical channel of the biosensor, and the transfer curves were monitored for 1 h. Each sample was tested on different devices to prevent cross-contamination.

**Figure 4:**
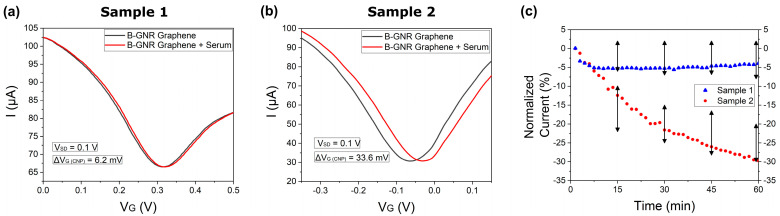
Transfer curves from exposure of the biosensor to human sera for 1 h. (**a**) A rightward shift in the transfer curve after exposing the biosensor to human serum that did not contain IgG antibodies (Serological Sample 1). (**b**) A rightward shift in the transfer curve after exposing the biosensor to human serum containing IgG antibodies (Serological Sample 2). (**c**) The current normalization of transfer curves A (blue) and B (red), as well as the black arrows, shows the range of results obtained from 10 different serological samples.

Figure 4a illustrates the transfer curves both before (black line) and after (red line) exposure to the serological sample 1. A slight shift in the charge neutrality point potential (ΔV_G (CNP)_) by 6.2 mV is observed here. Figure 4b replicates the test conducted in the previous figure, but this time, with sample 2. In this instance, the ΔV_G (CNP)_ variation is considerably more significant, yielding a result of 33.6 mV. Figure 4c shows a time versus normalized current plot involving sera samples 1 and 2. The normalization of current was conducted similarly: 0.1 V to the right of the CNP. This experiment was replicated for ten distinct serological samples, and the error bars within the figure indicate a distinction between the two groups of results. The entire plot of all 10 human sera is shown in Figure S8. The outcomes illustrate that, within one hour, four samples exhibited a current variation under 8%, which we classified as a negative sample, while six samples showcased a current variation surpassing 20%, which we classified as a positive sample. The same graph also highlights the capability to differentiate samples with or without IgG antibodies in less than 15 min.

In antigen–antibody binding assays involving serological samples, a blocker is typically used to prevent nonspecific proteins in the serum from binding to the antigen [57,58]. In our study, however, the specific antigen–antibody interaction generated a strong signal without the need for a blocker, streamlining the sensor manufacturing process by removing an extra step. Nevertheless, this resulted in longer detection times due to the abundance of nonspecific molecules in human serum, which quickly occupied the unmodified areas of the graphene surface. The significant noise caused by these nonspecific interactions ultimately delayed detection. This is demonstrated in Figure S9, which illustrates the results from when the antibodies from Sample 10, shown in Figure S8, were tested on both pristine graphene and graphene after B-GNR deposition. In the pristine graphene curve, a 5% current shift is observed, likely due to nonspecific interactions with the antibodies.

Table 1 compares the results of this study with previous diagnoses obtained through ELISA, demonstrating that our biosensor achieves a 90% accuracy rate relative to the gold-standard tests for COVID-19 antibody detection. Moreover, these results highlight the high selectivity of the B-GNR graphene sensor, as human sera contain a diverse range of IgG antibodies from past infections. Notably, as evidenced by comparison with ELISA, our sensor predominantly responded to IgG antibodies specific to SARS-CoV-2, with minimal cross-reactivity to antibodies from other infections. The sample names, results, and classifications from the ELISA assay were sourced from reference [12]. These findings underscore that our sensor provides results comparable to ELISA, while offering advantages such as ease of use, rapid detection, and greater cost-effectiveness. See Table 1.

In conclusion, this work presented promising results by applying nanostructured platforms, composed of graphene and gold nanorods functionalized with N-protein, to detect COVID-19. The measurements showed that the interaction between the antigen and antibody generated p-type doping in graphene, which was fundamental for detecting specific IgG, with the detection limit reaching up to 100 pg/mL. Clinical tests involving serological samples found that the current suffered a more significant disturbance with positive serum, and differentiation between positive and negative samples could be achieved in less than 15 min. In addition, this biosensor presented a 90% success rate in detecting the IgG antibody (anti-N-protein) for COVID-19, in relation to the gold-standard detection test, ELISA. Hence, our sensing platform holds the potential to be adapted to different pathogens, featuring a straightforward fabrication method, user-friendly operation, and rapid detection capabilities.

## Figures and Tables

**Table 1:** Comparative results of 10 different samples, analyzed in this work and by ELISA.

Sample Name (This Work)	Sample Name (ELISA)	Results (This Work) [*]	Results (ELISA) [a.u.]	Classification (This Work)	Classification (ELISA)
1	SCT 14	−3.7	0.4	Negative	Negative
2	SPF 139	−31.2	8.4	Positive	Positive
3	SCT 18	2.1	0.2	Negative	Negative
4	SPF 214	−26.5	1.5	Positive	Positive
**5**	**SCT 12**	**−22.2**	**0.5**	**Positive**	**Negative**
6	SPF 070	−25.3	5.4	Positive	Positive
7	SCT 13	−6.7	0.3	Negative	Negative
8	SPF 220	−28.4	7.4	Positive	Positive
9	SCT 11	−3.4	0.4	Negative	Negative
10	SPF 179	−28.7	6.1	Positive	Positive

* Percentage variation in normalized current over time.

## Data Availability

The raw data supporting the conclusions of this article will be made available by the authors upon request.

## Supplementary Materials

The following supporting information can be downloaded at https://www.mdpi.com/article/10.3390/bios15030164/s1. Supplementary Data S1, Supplementary Data S2, Supplementary Data S3. Figure S1: Transmission Electron Microscopy (TEM) images of gold nanorods (GNR). The left panel shows well-dispersed GNR, while the right panel presents a higher concentration of GNR, highlighting their morphology and distribution. Figure S2: Zeta potential analysis of B-GNR. GNR exhibited a zeta potential of +55 mV, indicating strong electrostatic repulsion and, consequently, high colloidal stability. With the addition of MUA, the zeta potential decreased to +50 mV, suggesting successful ligand adsorption on the surface without significantly affecting suspension stability. Finally, the introduction of N-protein further reduced the zeta potential to +47 mV, confirming successful bioconjugation while maintaining a sufficiently positive charge to prevent aggregation, thus preserving the biosensor’s functionality. Figure S3: Scanning Electron Microscope (SEM) images of the same sample device reveal B-GNR deposition on graphene, visible as white dots. In Figure S1c, the white contrast highlights the tips of the gold/chromium electrical contacts. Figure S1a–c shows a single deposition of gold nanorods on graphene via spin coating. Figure S1d–f depict a second deposition on the same spot as the images above. Figure S4: Illustration of the I × t plot construction. First, the gate voltage corresponding to the charge neutrality point (CNP) in the initial transfer curve was identified. Then, 0.1 V was added to this value, and the corresponding current at this voltage was monitored over time (dashed line), resulting in the I × t plot shown in the right panel. Figure S5: Schematic illustration of bioconjugated gold nanorods (B-GNRs). It is important to note that the elements are not drawn to scale, as the MUA and CTAB molecules are significantly smaller than the proteins and antibodies, by several orders of magnitude. Figure S6: Transfer curves representing the final curve (after 16 min) for each concentration of IgG monoclonal antibody deposition. Figure S7: This figure displays three applications of PBS and two applications of a monoclonal antibody (100 pg/mL) on bioconjugated graphene. The results clearly show that the addition of PBS does not affect the signal, in contrast to the response observed with the monoclonal antibody drop. Figure S8: This figure presents the complete plot of all 10 human sera from Figure 4c, demonstrating that within less than 15 min, it is possible to differentiate between sera with and without the presence of the IgG antibody. Figure S9: Transfer curves were obtained from the biosensor’s exposure to identical human serum samples for 1 h. Antibodies from Sample 10 in Figure S4 were tested both on pristine graphene (**a**) and on graphene following B-GNR deposition (**b**).

## References

[1] Zhou P., Yang X.-L., Wang X.-G., Hu B., Zhang L., Zhang W., Si H.-R., Zhu Y., Li B., Huang C.-L. (2020). A pneumonia outbreak associated with a new coronavirus of probable bat origin. Nature.

[2] World Health Organization (2020). WHO Director-General’s Opening Remarks at the Media Briefing on COVID-19—11 March 2020. https://www.who.int/director-general/speeches/detail/who-director-general-s-opening-remarks-at-the-media-briefing-on-covid-19---11-march-2020.

[3] Centers for Disease Control and Prevention (CDC/USA) (2022). Symptoms of COVID-19. https://www.cdc.gov/covid/signs-symptoms/?CDC_AAref_Val=https://www.cdc.gov/coronavirus/2019-ncov/symptoms-testing/symptoms.

[4] Centers for Disease Control and Prevention (CDC/USA) (2024). Infection Control Guidance: SARS-CoV-2. https://www.cdc.gov/covid/hcp/infection-control/?CDC_AAref_Val=https://www.cdc.gov/coronavirus/2019-ncov/hcp/infection-control-recommendations.html.

[5] Kumar S., Nyodu R., Maurya V.K., Saxena S.K. (2020). Morphology, Genome Organization, Replication, and Pathogenesis of Severe Acute Respiratory Syndrome Coronavirus 2 (SARS-CoV-2). Medical Virology: From Pathogenesis to Disease Control.

[6] Maghsood F., Ghorbani A., Yadegari H., Golsaz-Shirazi F., Amiri M.M., Shokri F. (2023). SARS-CoV-2 nucleocapsid: Biological functions and implication for disease diagnosis and vaccine design. Rev. Med. Virol..

[7] Alvim R.G., Lima T.M., Rodrigues D.A., Marsili F.F., Bozza V.B., Higa L.M., Monteiro F.L., Abreu D.P., Leitão I.C., Carvalho R.S. (2022). From a recombinant key antigen to an accurate, affordable serological test: Lessons learnt from COVID-19 for future pandemics. Biochem. Eng. J..

[8] Caddy S.L., Vaysburd M., Papa G., Wing M., O’connell K., Stoycheva D., Foss S., Andersen J.T., Oxenius A., James L.C. (2021). Viral nucleoprotein antibodies activate TRIM21 and induce T cell immunity. EMBO J..

[9] Okba N.M.A., Müller M.A., Li W., Wang C., GeurtsvanKessel C.H., Corman V.M., Lamers M.M., Sikkema R.S., De Bruin E., Chandler F.D. (2020). Severe Acute Respiratory Syndrome Coronavirus 2−Specific Antibody Responses in Coronavirus Disease Patients. Emerg. Infect. Dis..

[10] Perera R.A., Mok C.K., Tsang O.T., Lv H., Ko R.L., Wu N.C., Yuan M., Leung W.S., MC Chan J., Chik T.S. (2020). Serological assays for severe acute respiratory syndrome coronavirus 2 (SARS-CoV-2), March 2020. Euro Surveill..

[11] Ludolf F., Ramos F.F., Bagno F.F., Oliveira-Da-Silva J.A., Reis T.A.R., Christodoulides M., Vassallo P.F., Ravetti C.G., Nobre V., da Fonseca F.G. (2022). Detecting anti–SARS-CoV-2 antibodies in urine samples: A noninvasive and sensitive way to assay COVID-19 immune conversion. Sci. Adv..

[12] Bagno F.F., Sérgio S.A., Figueiredo M.M., Godoi L.C., Andrade L.A., Salazar N.C., Soares C.P., Aguiar A., Almeida F.J., da Silva E.D. (2022). Development and validation of an enzyme-linked immunoassay kit for diagnosis and surveillance of COVID-19. J. Clin. Virol. Plus.

[13] Sakamoto S., Putalun W., Vimolmangkang S., Phoolcharoen W., Shoyama Y., Tanaka H., Morimoto S. (2018). Enzyme-linked immunosorbent assay for the quantitative/qualitative analysis of plant secondary metabolites. J. Nat. Med..

[14] Human SARS-CoV-2 Spike (Trimer) IgG ELISA Kit. ThermoFisher Scientific. https://www.thermofisher.com/elisa/product/Human-SARS-CoV-2-Spike-Trimer-IgG-ELISA-Kit/BMS2325.

[15] Ang G., Chan K., Yean C., Yu C. (2022). Lateral Flow Immunoassays for SARS-CoV-2. Diagnostics.

[16] Li Z., Yi Y., Luo X., Xiong N., Liu Y., Li S., Sun R., Wang Y., Hu B., Chen W. (2020). Development and clinical application of a rapid IgM-IgG combined antibody test for SARS-CoV-2 infection diagnosis. J. Med. Virol..

[17] Geim A.K., Novoselov K.S. (2007). The rise of graphene. Nat. Mater..

[18] Abergel D.S.L., Apalkov V., Berashevich J., Ziegler K., Chakraborty T. (2010). Properties of graphene: A theoretical perspective. Adv. Phys..

[19] Lee C., Wei X., Kysar J.W., Hone J. (2008). Measurement of the Elastic Properties and Intrinsic Strength of Monolayer Graphene. Science.

[20] Béraud A., Sauvage M., Bazán C.M., Tie M., Bencherif A., Bouilly D. (2021). Graphene field-effect transistors as bioanalytical sensors: Design, operation and performance. Analyst.

[21] Halder A., Zhang M., Chi Q. (2017). Electroactive and biocompatible functionalization of graphene for the development of biosensing platforms. Biosens. Bioelectron..

[22] Matsumoto K., Maehashi K., Ohno Y., Inoue K. (2014). Recent advances in functional graphene biosensors. J. Phys. D Appl. Phys..

[23] Bai S., Shen X. (2012). Graphene–inorganic nanocomposites. RSC Adv..

[24] Turcheniuk K., Boukherroub R., Szunerits S. (2015). Gold–graphene nanocomposites for sensing and biomedical applications. J. Mater. Chem. B.

[25] Mao S., Lu G., Yu K., Bo Z., Chen J. (2010). Specific Protein Detection Using Thermally Reduced Graphene Oxide Sheet Decorated with Gold Nanoparticle-Antibody Conjugates. Adv. Mater..

[26] Abraham S., Nirala N.R., Pandey S., Srivastava M., Srivastava S., Walkenfort B., Srivastava A. (2015). Functional graphene–gold nanoparticle hybrid system for enhanced electrochemical biosensing of free cholesterol. Anal. Methods.

[27] Cai B., Wang S., Huang L., Ning Y., Zhang Z., Zhang G.-J. (2014). Ultrasensitive Label-Free Detection of PNA–DNA Hybridization by Reduced Graphene Oxide Field-Effect Transistor Biosensor. ACS Nano.

[28] Lerner M.B., Matsunaga F., Han G.H., Hong S.J., Xi J., Crook A., Perez-Aguilar J.M., Park Y.W., Saven J.G., Liu R. (2014). Scalable Production of Highly Sensitive Nanosensors Based on Graphene Functionalized with a Designed G Protein-Coupled Receptor. Nano Lett..

[29] Dong X., Shi Y., Huang W., Chen P., Li L. (2010). Electrical Detection of DNA Hybridization with Single-Base Specificity Using Transistors Based on CVD-Grown Graphene Sheets. Adv. Mater..

[30] Ambrosi A., Chua C.K., Bonanni A., Pumera M. (2014). Electrochemistry of Graphene and Related Materials. Chem. Rev..

[31] Szunerits S., Maalouli N., Wijaya E., Vilcot J.-P., Boukherroub R. (2013). Recent advances in the development of graphene-based surface plasmon resonance (SPR) interfaces. Anal. Bioanal. Chem..

[32] Gil B., Keshavarz M., Wales D., Darzi A., Yeatman E. (2023). Orthogonal Surface-Enhanced Raman Scattering/Field-Effect Transistor Detection of Breast and Colorectal Cancer-Derived Exosomes using Graphene as a Tag-Free Diagnostic Template. Adv. NanoBiomed. Res..

[33] Viswanathan S., Narayanan T.N., Aran K., Fink K.D., Paredes J., Ajayan P.M., Filipek S., Miszta P., Tekin H.C., Inci F. (2015). Graphene–protein field effect biosensors: Glucose sensing. Mater. Today.

[34] Szunerits S., Rodrigues T., Bagale R., Happy H., Boukherroub R., Knoll W. (2023). Graphene-based field-effect transistors for biosensing: Where is the field heading to?. Anal. Bioanal. Chem..

[35] Cui T.-R., Qiao Y.-C., Gao J.-W., Wang C.-H., Zhang Y., Han L., Yang Y., Ren T.-L. (2021). Ultrasensitive Detection of COVID-19 Causative Virus (SARS-CoV-2) Spike Protein Using Laser Induced Graphene Field-Effect Transistor. Molecules.

[36] Shahdeo D., Chauhan N., Majumdar A., Ghosh A., Gandhi S. (2022). Graphene-Based Field-Effect Transistor for Ultrasensitive Immunosensing of SARS-CoV-2 Spike S1 Antigen. ACS Appl. Bio Mater..

[37] Silvestri A., Zayas-Arrabal J., Vera-Hidalgo M., Di Silvio D., Wetzl C., Martinez-Moro M., Zurutuza A., Torres E., Centeno A., Maestre A. (2023). Ultrasensitive detection of SARS-CoV-2 spike protein by graphene field-effect transistors. Nanoscale.

[38] Park I., Lim J., You S., Hwang M.T., Kwon J., Koprowski K., Kim S., Heredia J., de Ramirez S.A.S., Valera E. (2021). Detection of SARS-CoV-2 Virus Amplification Using a Crumpled Graphene Field-Effect Transistor Biosensor. ACS Sens..

[39] Gao J., Wang C., Chu Y., Han Y., Gao Y., Wang Y., Wang C., Liu H., Han L., Zhang Y. (2022). Graphene oxide-graphene Van der Waals heterostructure transistor biosensor for SARS-CoV-2 protein detection. Talanta.

[40] Kim S., Ryu H., Tai S., Pedowitz M., Rzasa J.R., Pennachio D.J., Hajzus J.R., Milton D.K., Myers-Ward R., Daniels K.M. (2022). Real-time ultra-sensitive detection of SARS-CoV-2 by quasi-freestanding epitaxial graphene-based biosensor. Biosens. Bioelectron..

[41] Seo G., Lee G., Kim M.J., Baek S.-H., Choi M., Ku K.B., Lee C.-S., Jun S., Park D., Kim H.G. (2020). Rapid Detection of COVID-19 Causative Virus (SARS-CoV-2) in Human Nasopharyngeal Swab Specimens Using Field-Effect Transistor-Based Biosensor. ACS Nano.

[42] Kang H., Wang X., Guo M., Dai C., Chen R., Yang L., Wu Y., Ying T., Zhu Z., Wei D. (2021). Ultrasensitive Detection of SARS-CoV-2 Antibody by Graphene Field-Effect Transistors. Nano Lett..

[43] Liu H., Yang A., Song J., Wang N., Lam P., Li Y., Law H.K.-W., Yan F. (2021). Ultrafast, sensitive, and portable detection of COVID-19 IgG using flexible organic electrochemical transistors. Sci. Adv..

[44] Mattioli I.A., Castro K.R., Macedo L.J., Sedenho G.C., Oliveira M.N., Todeschini I., Vitale P.M., Ferreira S.C., Manuli E.R., Pereira G.M. (2022). Graphene-based hybrid electrical-electrochemical point-of-care device for serologic COVID-19 diagnosis. Biosens. Bioelectron..

[45] Torrente-Rodríguez R.M., Lukas H., Tu J., Min J., Yang Y., Xu C., Rossiter H.B., Gao W. (2020). SARS-CoV-2 RapidPlex: A Graphene-Based Multiplexed Telemedicine Platform for Rapid and Low-Cost COVID-19 Diagnosis and Monitoring. Matter.

[46] Novodchuk I., Kayaharman M., Prassas I., Soosaipillai A., Karimi R., Goldthorpe I., Abdel-Rahman E., Sanderson J., Diamandis E., Bajcsy M. (2022). Electronic field effect detection of SARS-CoV-2 N-protein before the onset of symptoms. Biosens. Bioelectron..

[47] Pereira C.L., Cadore A., Rezende N.P., Gadelha A., Soares E.A., Chacham H., Campos L.C., Lacerda R.G. (2019). Reversible doping of graphene field effect transistors by molecular hydrogen: The role of the metal/graphene interface. 2D Mater..

[48] Alves A.P.P., Meireles L.M., Ferrari G.A., Cunha T.H.R., Paraense M.O., Campos L.C., Lacerda R.G. (2020). Highly sensitive and reusable ion-sensor based on functionalized graphene. Appl. Phys. Lett..

[49] Silvestre I., de Morais E.A., Melo A.O., Campos L.C., Goncalves A.-M.B., Cadore A.R., Ferlauto A.S., Chacham H., Mazzoni M.S.C., Lacerda R.G. (2013). Asymmetric Effect of Oxygen Adsorption on Electron and Hole Mobilities in Bilayer Graphene: Long- and Short-Range Scattering Mechanisms. ACS Nano.

[50] Yu C., Irudayaraj J. (2006). Multiplex Biosensor Using Gold Nanorods. Anal. Chem..

[51] Chu C.-H., Sarangadharan I., Regmi A., Chen Y.-W., Hsu C.-P., Chang W.-H., Lee G.-Y., Chyi J.-I., Chen C.-C., Shiesh S.-C. (2017). Beyond the Debye length in high ionic strength solution: Direct protein detection with field-effect transistors (FETs) in human serum. Sci. Rep..

[52] Casiraghi C., Pisana S., Novoselov K.S., Geim A.K., Ferrari A.C. (2007). Raman fingerprint of charged impurities in graphene. Appl. Phys. Lett..

[53] Das A., Pisana S., Chakraborty B., Piscanec S., Saha S.K., Waghmare U.V., Novoselov K.S., Krishnamurthy H.R., Geim A.K., Ferrari A.C. (2008). Monitoring dopants by Raman scattering in an electrochemically top-gated graphene transistor. Nat. Nanotechnol..

[54] Ferrari A.C., Meyer J.C., Scardaci V., Casiraghi C., Lazzeri M., Mauri F., Piscanec S., Jiang D., Novoselov K.S., Roth S. (2006). Raman Spectrum of Graphene and Graphene Layers. Phys. Rev. Lett..

[55] Liu H., Liu Y., Zhu D. (2011). Chemical doping of graphene. J. Mater. Chem..

[56] Prado M.C., Nascimento R., Moura L.G., Matos M.J.S., Mazzoni M.S.C., Cancado L.G., Chacham H., Neves B.R.A. (2011). Two-Dimensional Molecular Crystals of Phosphonic Acids on Graphene. ACS Nano.

[57] Lin Y., Liu K., Wang C., Li L., Liu Y. (2015). Electrochemical Immunosensor for Detection of Epidermal Growth Factor Reaching Lower Detection Limit: Toward Oxidized Glutathione as a More Efficient Blocking Reagent for the Antibody Functionalized Silver Nanoparticles and Antigen Interaction. Anal. Chem..

[58] Martín-Yerga D., Costa-García A. (2015). Towards a blocking-free electrochemical immunosensing strategy for anti-transglutaminase antibodies using screen-printed electrodes. Bioelectrochemistry.

