# Supplementary material for: Detection of IgG Antibodies Against COVID-19 N-Protein by Hybrid Graphene–Nanorod Sensor
Source: Biosensors (Basel). 2025 Mar 4;15(3):164. doi: 10.3390/bios15030164 (PMC11940001; doi:10.3390/bios15030164)
Supplement: Supplementary file 1 [file biosensors-15-00164-s001.zip › biosensors-3485351-supplementary.pdf]

biosensors

Supplementary Materials

Detection of IgG antibody against COVID-19 N-protein by a Hybrid Graphene-Nanorod sensor

R. V. A. Boaventura[1,2]; C. L. Pereira[1]; C. Junqueira[2,3]; K. B. Gonçalves[1,2]; N. P. Rezende[1]; I. B. Apolinário[2]; R. C. Barcelos[4]; F. B. Oréfice[1,2]; F. F. Bagno[5]; F. G. Fonseca[3]; A. Corrêa Jr[2,3]; L. S. Gomes[1,2]; R. G. Lacerda[1,2*]

[1] Physics Departament, Federal University of Minas Gerais (UFMG), Belo Horizonte, MG, Brazil.
[2] CTNano, Federal University of Minas Gerais (UFMG), Belo Horizonte, MG, Brazil.
[3] Microbiology Department, Federal University of Minas Gerais (UFMG), Belo Horizonte, MG, Brazil.
[4] Chemistry Department, Federal University of São João del-Rei (UFSJ), Divinópolis, MG, Brazil.
[5] Vaccine Technology Center (CT Vacinas), BH-Tec, UFMG. Belo Horizonte, MG, Brazil.
* Correspondence: rlacerda@fisica.ufmg.br;

Supplementary Data S1

To perform the bioconjugation process, a concentration/amount of protein N is added, considering the number of available binding sites present on the GNRs, mediated by the linker MUA (mercaptoundecanoic acid). This linker facilitates the connection between protein N and the GNR surface through specific chemical bonds, promoting a stable attachment. At the end of the bioconjugation process, the unbound protein N is removed through successive washing steps using appropriate buffers to maintain the conformation and stability of both the protein N and the GNRs. This purification process is essential to eliminate free proteins, preventing interference in subsequent steps and ensuring the effectiveness of the final bioconjugate.

The nucleocapsid protein (N) used in this study, according to the GenBank accession sequence MT126808.1, is composed of 419 amino acids, and its molecular mass is estimated to be around 45-50 kDa. This protein was expressed in its full form, ensuring that all relevant functions of the protein, including its interaction with RNA and its ability to form the nucleocapsid, were maintained. The complete nucleocapsid protein (N) gene region of SARS-CoV-2 was codon-optimized and inserted into the pET-24a-(+) expression vector, later used to transform the E. coli BL21(DE3) strain. The recombinant protein was purified through affinity chromatography on nickel columns using the AKTAprime plus system, according to the manufacturer's instructions (GE Healthcare, USA).

Supplementary Data S2

Scanning Electron Microscope (SEM) images were captured to verify the deposition of B-GNRs, as shown in figure S1. The images were obtained using a secondary electron detector with an electron high tension (EHT) of 20 kV under high vacuum conditions. To quantitatively assess the distribution of GNRs on graphene, the surface coverage was calculated by analyzing the area occupied by the nanorods relative to the total scanned area. Figure S1 (a, b, and c) serve as representative samples, showing an arithmetic average concentration of B-GNRs on graphene of (6.82 ± 1.77) B-GNR/μm^2, with the uncertainty representing the standard deviation. A typical mechanical exfoliation graphene has size of approximately 20μm x 20 μm, so in this area, the arithmetic average of B-GNRs on graphene is (2726 ± 707) GNR/graphene. After a wash step, an additional deposition of B-GNRs on graphene was made, as shown in figure S1 (d, e, and f), at the exact same location as the previous panels. This demonstrates the strong binding affinity between graphene and B-GNRs, as the particles from the first deposition remained in the same spot and orientation after washing, redeposition, and spin coating.

Supplementary Data S3

The Ag/AgCl commercial electrode used in this study was the ET072-3 model from eDAQ. This electrode has a length of 65 mm, a diameter of 2 mm, and a metal connector with a 1 mm diameter. Its body is made of PEEK (polyetheretherketone), which is highly resistant to most organic solvents. The electrode has an impedance of less than 10 kΩ and is ideal for electrochemistry research. Additionally, it is completely leak-proof, ensuring that samples are not contaminated by chloride or silver ions.

Before each experiment, we conducted a few transfer curves using PBS over a test sample to verify the electrode's condition. If the transfer curves were noisier than in previous tests, we cleaned the electrode by immersing it in a strong acid solution (6 mol/L H_2SO_4) for 30 minutes, followed by 10 minutes of sonication in deionized water. This cleaning method effectively removed any protein adsorbed on the electrode surface.

(a) (b)

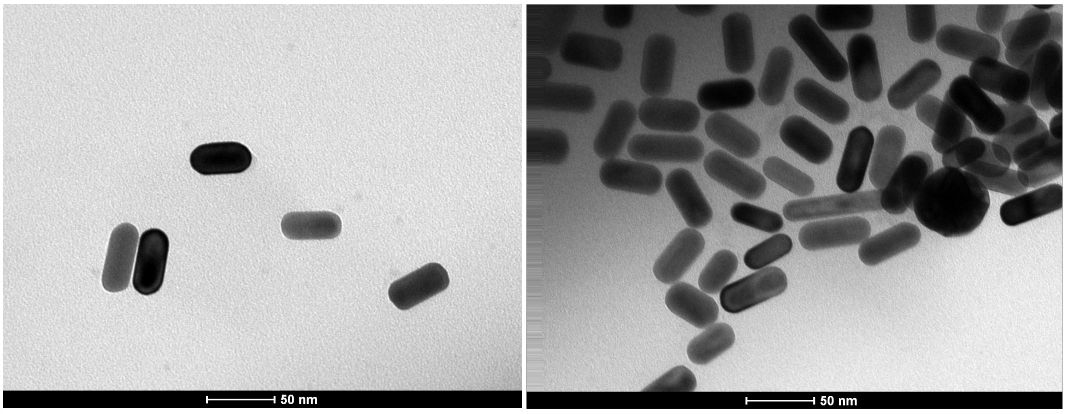

Figure S1. Transmission Electron Microscopy (TEM) images of gold nanorods (GNR). The left panel shows well-dispersed GNR, while the right panel presents a higher concentration of GNR, highlighting their morphology and distribution.

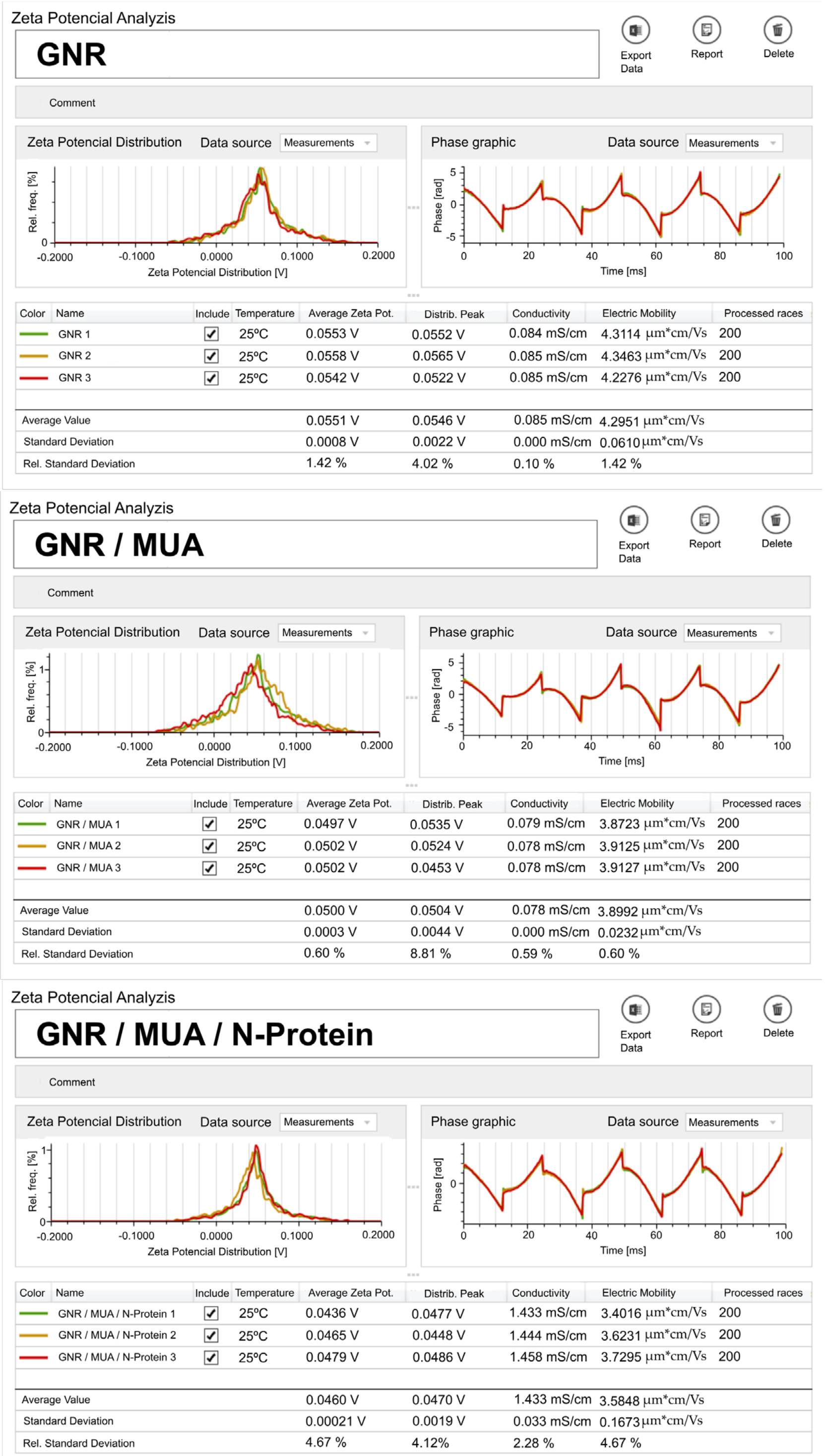

GNR

Color	Name	Include	Temperature	Average Zeta Pot.	Distrib. Peak	Conductivity	Electric Mobility	Processed races
green	GNR 1	☑	25°C	0.0553 V	0.0552 V	0.084 mS/cm	4.3114 µm*cm/Vs	200
orange	GNR 2	☑	25°C	0.0558 V	0.0565 V	0.085 mS/cm	4.3463 µm*cm/Vs	200
red	GNR 3	☑	25°C	0.0542 V	0.0522 V	0.085 mS/cm	4.2276 µm*cm/Vs	200
	Average Value			0.0551 V	0.0546 V	0.085 mS/cm	4.2951 µm*cm/Vs	
	Standard Deviation			0.0008 V	0.0022 V	0.000 mS/cm	0.0610 µm*cm/Vs	
	Rel. Standard Deviation			1.42 %	4.02 %	0.10 %	1.42 %	

GNR / MUA

Color	Name	Include	Temperature	Average Zeta Pot.	Distrib. Peak	Conductivity	Electric Mobility	Processed races
green	GNR / MUA 1	☑	25°C	0.0497 V	0.0535 V	0.079 mS/cm	3.8723 µm*cm/Vs	200
orange	GNR / MUA 2	☑	25°C	0.0502 V	0.0524 V	0.078 mS/cm	3.9125 µm*cm/Vs	200
red	GNR / MUA 3	☑	25°C	0.0502 V	0.0453 V	0.078 mS/cm	3.9127 µm*cm/Vs	200
	Average Value			0.0500 V	0.0504 V	0.078 mS/cm	3.8992 µm*cm/Vs	
	Standard Deviation			0.0003 V	0.0044 V	0.000 mS/cm	0.0232 µm*cm/Vs	
	Rel. Standard Deviation			0.60 %	8.81 %	0.59 %	0.60 %	

GNR / MUA / N-Protein

Color	Name	Include	Temperature	Average Zeta Pot.	Distrib. Peak	Conductivity	Electric Mobility	Processed races
green	GNR / MUA / N-Protein 1	☑	25°C	0.0436 V	0.0477 V	1.433 mS/cm	3.4016 µm*cm/Vs	200
orange	GNR / MUA / N-Protein 2	☑	25°C	0.0465 V	0.0448 V	1.444 mS/cm	3.6231 µm*cm/Vs	200
red	GNR / MUA / N-Protein 3	☑	25°C	0.0479 V	0.0486 V	1.458 mS/cm	3.7295 µm*cm/Vs	200
	Average Value			0.0460 V	0.0470 V	1.433 mS/cm	3.5848 µm*cm/Vs	
	Standard Deviation			0.00021 V	0.0019 V	0.033 mS/cm	0.1673 µm*cm/Vs	
	Rel. Standard Deviation			4.67 %	4.12%	2.28 %	4.67 %	

Figure S2. Zeta potential analysis of B-GNR. GNR exhibited a zeta potential of +55 mV, indicating strong electrostatic repulsion and, consequently, high colloidal stability. With the addition of MUA, the zeta potential decreased to +50 mV, suggesting successful ligand adsorption on the surface without significantly affecting suspension stability. Finally, the introduction of N-protein further reduced the zeta potential to +47 mV, confirming successful bioconjugation while maintaining a sufficiently positive charge to prevent aggregation, thus preserving the biosensor's functionality.

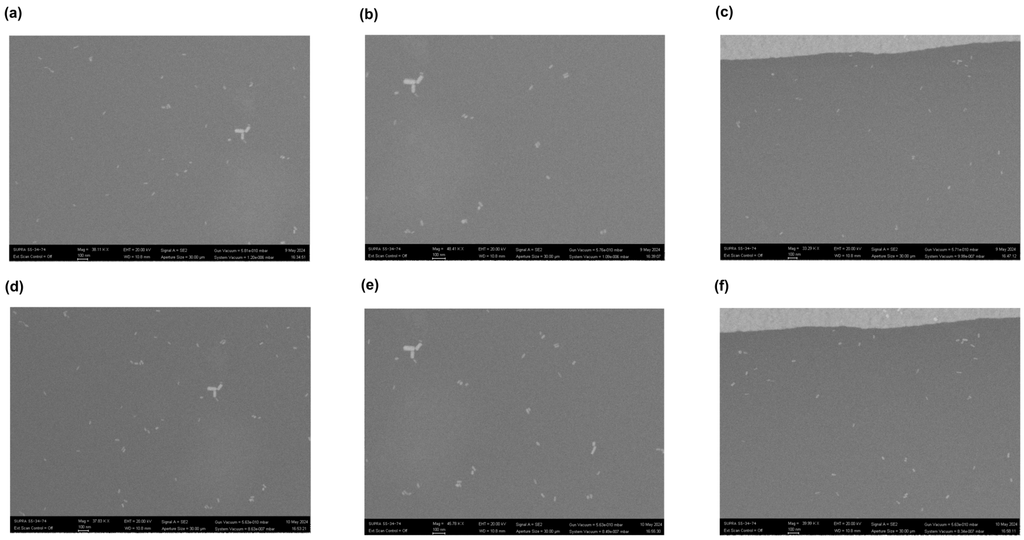

Figure S3. Scanning Electron Microscope (SEM) images of the same sample device reveal B-GNR deposition on graphene, visible as white dots. In figure S1(c), the white contrast highlights the tips of the gold/chromium electrical contacts. Figure S1 (**a**, **b**, and **c**) shows a single deposition of gold nanorods on graphene via spin coating. Figure S1 (**d**,**e**, and **f**) depict a second deposition on the same spot as the images above.

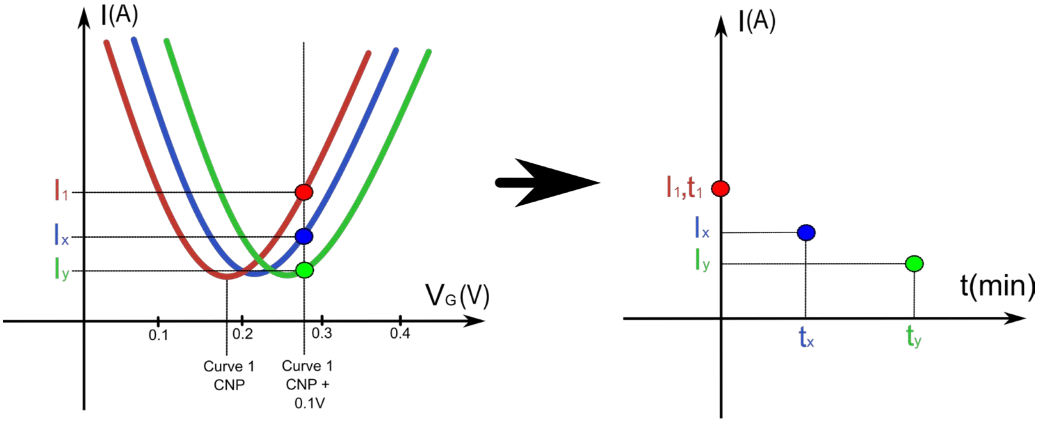

Figure S4. Illustration of the I × t plot construction. First, the gate voltage corresponding to the charge neutrality point (CNP) in the initial transfer curve was identified. Then, 0.1 V was added to this value, and the corresponding current at this voltage was monitored over time (dashed line), resulting in the I × t plot shown in the right panel.

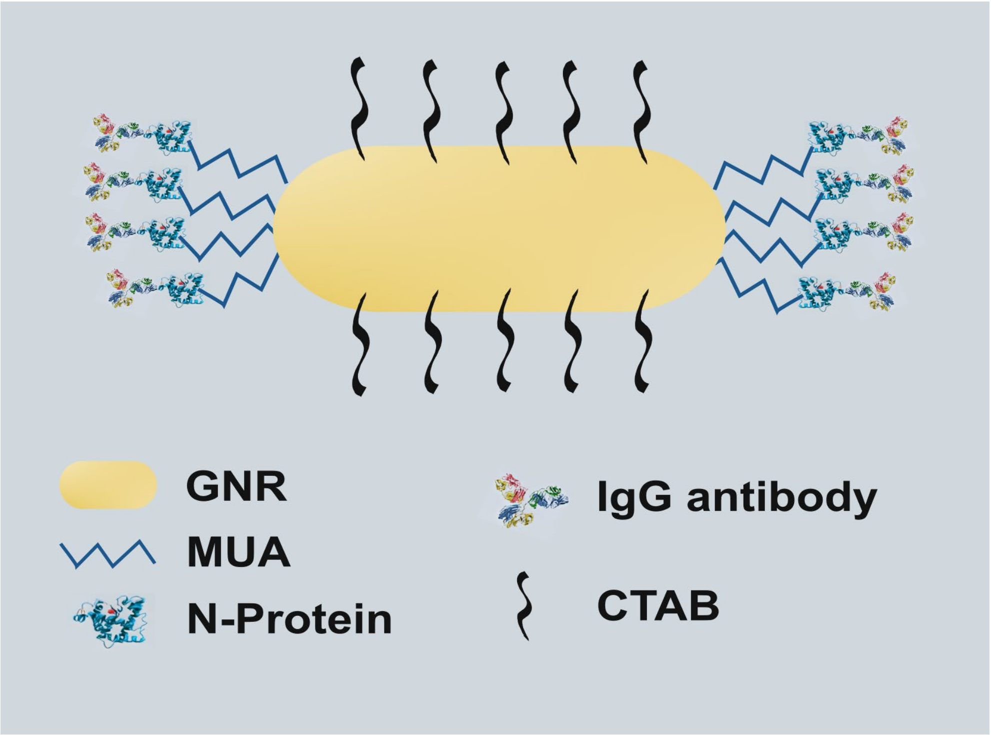

Figure S5. Schematic illustration of bioconjugated gold nanorods (B-GNRs). It is important to note that the elements are not drawn to scale, as the MUA and CTAB molecules are significantly smaller than the proteins and antibodies, by several orders of magnitude.

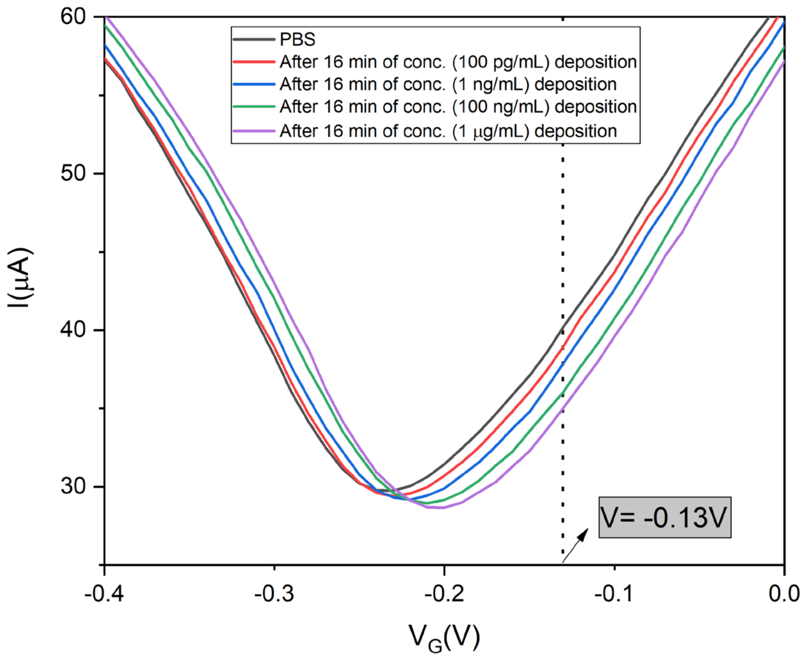

Figure S6. Transfer curves representing the final curve (after 16 minutes) for each concentration of IgG monoclonal antibody deposition.

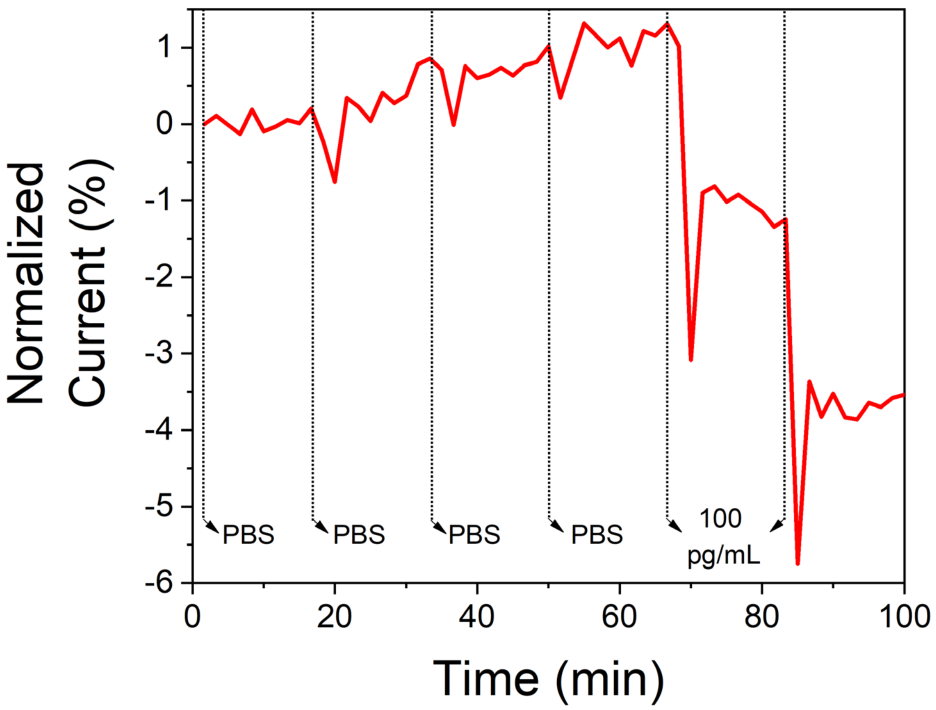

Figure S7. This figure displays three applications of PBS and two applications of a monoclonal antibody (100 pg/mL) on bioconjugated graphene. The results clearly show that the addition of PBS does not affect the signal, in contrast to the response observed with the monoclonal antibody drop.

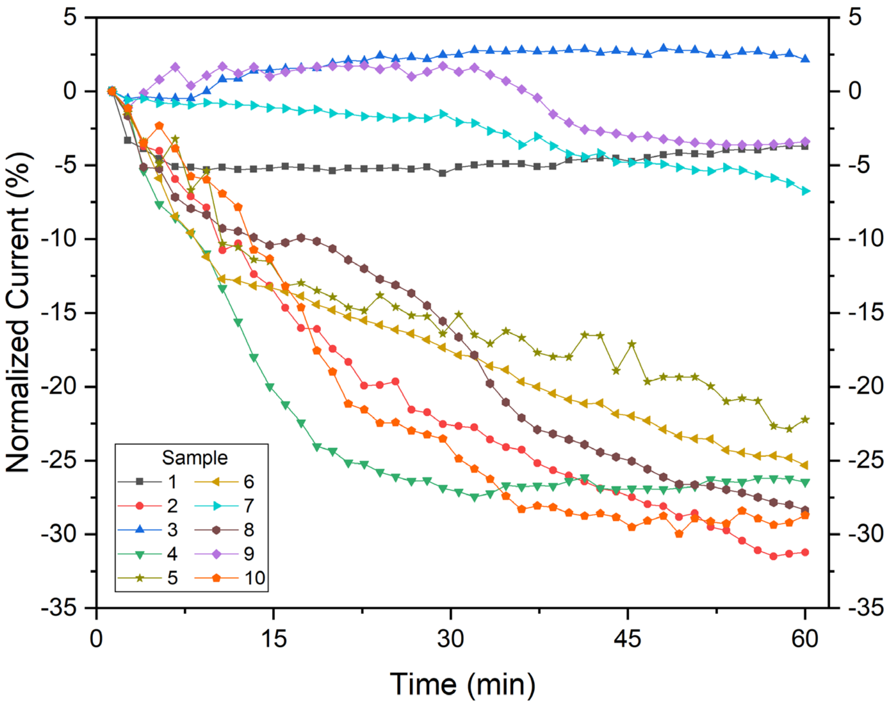

Figure S8. This figure presents the complete plot of all 10 human sera from figure 4(c), demonstrating that within less than 15 minutes, it is possible to differentiate between sera with and without the presence of the IgG antibody.

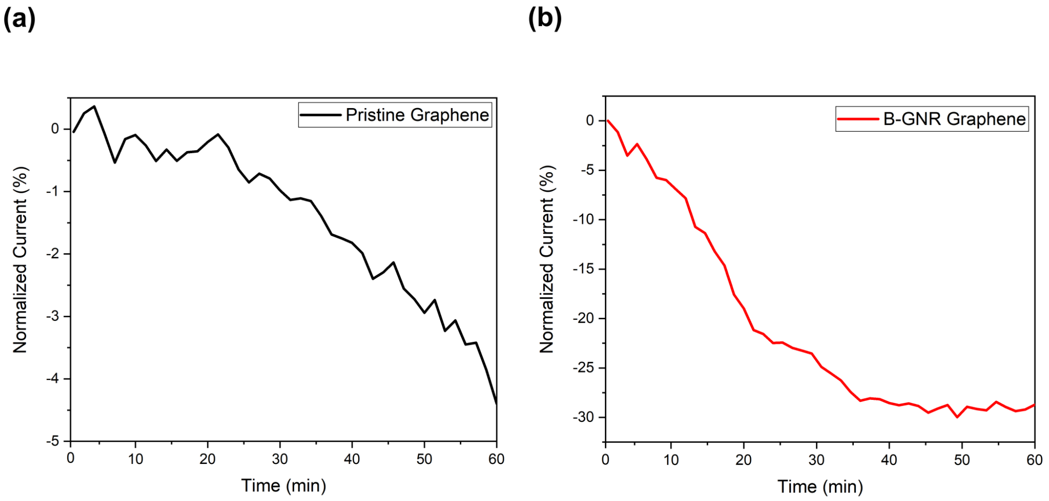

Figure S9. Transfer curves were obtained from the biosensor's exposure to identical human serum samples for 1 hour. Antibodies from Sample 10 in figure S4 were tested both on pristine graphene (**a**) and on graphene following B-GNR deposition (**b**).
